# Severe functional ischaemic mitral regurgitation: is functional a misnomer for a dysfunctional valve? A case report

**DOI:** 10.1093/ehjcr/ytae041

**Published:** 2024-01-30

**Authors:** Michael P Chrissoheris, Panagiota Kourkoveli, Dionysios Aravantinos, Konstantinos Spargias

**Affiliations:** Department of Transcatheter Heart Valves, Hygeia Hospital, 9 Erythrou Stavrou Street, Marousi, TK 15123 Attiki, Greece; Department of Transcatheter Heart Valves, Hygeia Hospital, 9 Erythrou Stavrou Street, Marousi, TK 15123 Attiki, Greece; Department of Transcatheter Heart Valves, Hygeia Hospital, 9 Erythrou Stavrou Street, Marousi, TK 15123 Attiki, Greece; Department of Transcatheter Heart Valves, Hygeia Hospital, 9 Erythrou Stavrou Street, Marousi, TK 15123 Attiki, Greece

**Keywords:** Functional mitral regurgitation, Transcatheter edge to edge repair, Heart failure device therapy, Case report

## Abstract

**Background:**

Mitral regurgitation (MR) in the context of left ventricular systolic dysfunction is often designated as functional, with emphasis on the underlying cardiomyopathy leading to malcoaptation of the ‘otherwise normal valve’.

**Case summary:**

A 63-year-old male with ischaemic cardiomyopathy (left ventricular ejection fraction 20%) presented with intractable heart failure in need of inotropic support and could not be stepped down from an ICU hospital setting. Functional MR, graded as moderate on transthoracic echocardiography, was initially not considered as pertinent to the clinical condition and options discussed included initiation of dialysis for volume management, chronic inotropic support, and palliative measures. However, a re-examination of the mitral valve by transoesophageal echo revealed severe regurgitation from annular dilatation and restricted mobility during systole. Transcatheter edge to edge repair utilizing the PASCAL device resulted in marked reduction of MR followed by an abrupt clinical improvement, weaning off inotropes and discharge home 4 days later. At four-year follow-up, the patient is stable on optimal heart failure therapy.

**Discussion:**

For many patients with heart failure and underlying cardiomyopathy, the presence of significant functional MR, instead of a ‘bystander’ disease, actually becomes the dominant driver of symptoms and compounds the low cardiac output state. In these patients, the term ‘functional’ MR becomes a misnomer, as in fact the so called ‘otherwise normal’ mitral valve is actually a severely dysfunctional valve with a wide malcoaptation zone. Transcatheter edge to edge repair is an effective bailout procedure for patients with low cardiac output and disproportionate severe functional MR.

**Learning points**

- The application of mitral transcatheter edge to edge repair (M-TEER) in a patient with decompensated heart failure unable to be stepped down from an ICU setting.
- The priority given to M-TEER for bailout, rather than proceed with the standard approach of cardiac resynchronization first, as would be the case in more stable and ambulatory patients.
- The possible use of the Pascal P10 device as a single device for an optimal outcome, as an alternative to using multiple MitraClips.
- The need to reconsider the term functional when describing secondary mitral regurgitation. For many, ‘functional’ often is interpreted as ‘benign’, but when it is of disproportionate severity, it can become as malignant as a destroyed valve.

## Introduction

For patients with ischaemic or dilated cardiomyopathy, New York Heart Association (NYHA) functional class and prognosis are closely associated with the degree of left ventricular systolic dysfunction, with any coexisting functional mitral regurgitation (FMR) often considered as a ‘bystander’ problem, a ‘normal’ valve that is not closing well due to tethering forces and annular dilatation. However, in many patients, it is this functional MR that becomes the dominant problem impacting heavily on symptoms and prognosis.^1–4^ Essentially, this ‘otherwise normal valve’ is in fact a dysfunctional valve, leading to severe regurgitation and intractable heart failure. The advent of transcatheter leaflet repair technologies has clearly identified functional MR to be a singular therapeutic target with major impact on clinical outcomes in well selected patients.^5^

## Summary figure

**Figure:**
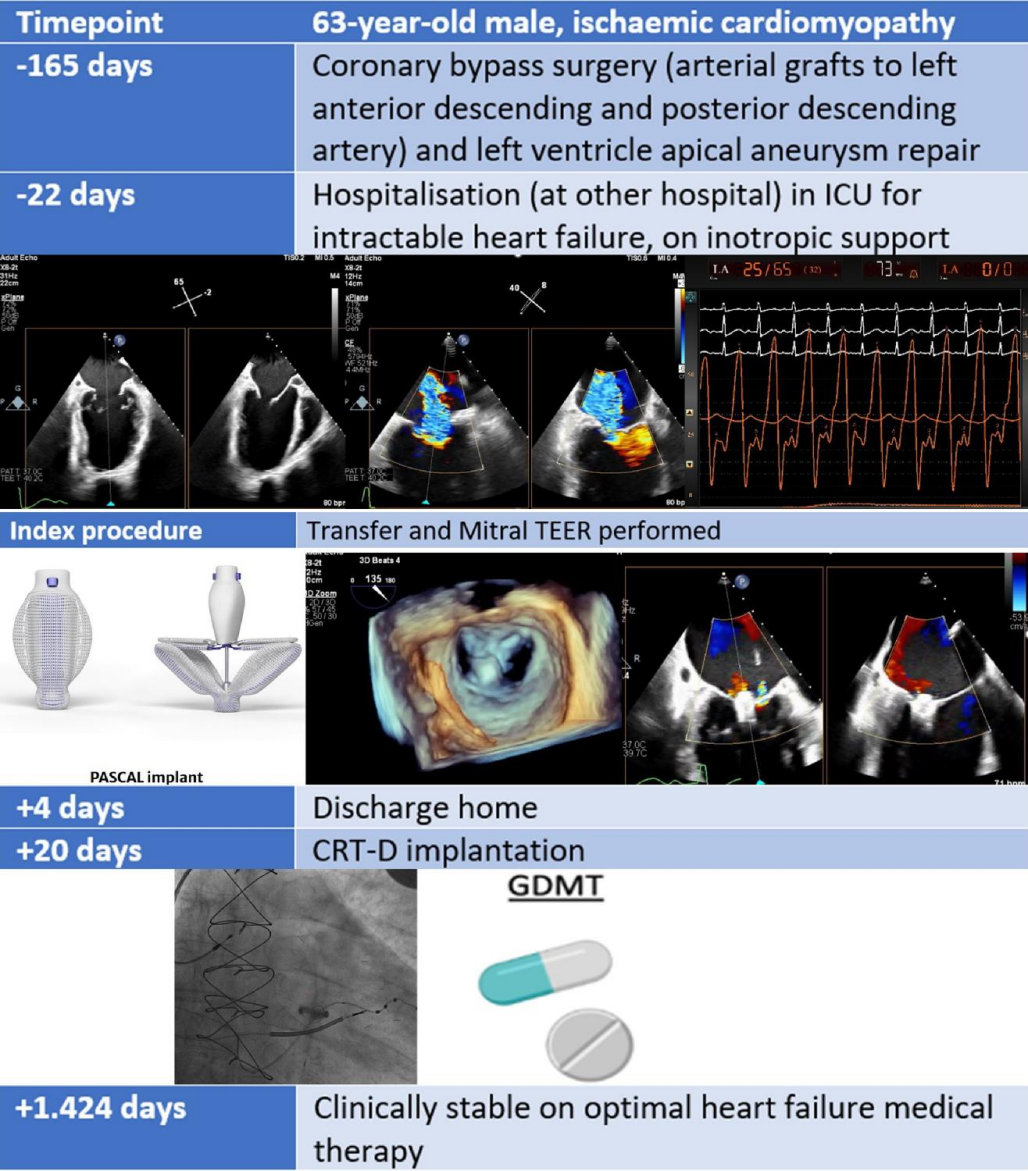


## Case presentation

A 63-year-old man with significant ischaemic FMR and NYHA functional class IV symptoms, on maximally tolerated medical therapy, was referred for inability to wean off inotropic support while in an intensive care unit (ICU) setting for several days (Interagency Registry for Mechanically Assisted Circulatory Support INTERMACS III)^6^ with clinical evidence of low cardiac output state (acute on chronic kidney failure, hepatic dysfunction, and respiratory failure). Upon transfer, he was confined to bed (for the previous 22 days), afebrile, with blood pressure of 100/60 mmHg, heart rate of 90 beats/min (on dobutamine infusion), and pulse oximetry of 95% on 40% FiO_2_ via face mask. Cardiovascular examination was notable for a 2/6 apical systolic murmur, with signs of decompensated heart failure (low cardiac output and volume overload).

The past medical history was notable for premature coronary artery disease and acute myocardial infarction at age 48 (conservative treatment). The most recent coronary angiogram revealed patent left main, occlusion of the mid left anterior descending, atheromatous left circumflex and right coronary with 70% stenosis of the posterior descending artery. Following this, surgical revascularization was performed (see *Summary figure*) with use of both internal mammaries as grafts, along with aneurysmectomy of the left ventricular apex. Other comorbidities included chronic kidney disease (maximum creatinine level of 3.0 mg/dL, estimated glomerular filtration rate 21 mL/min/1.73 m^2^) and obesity (body mass index 30.4 kg/m^2^).

The differential diagnosis for intractable heart failure was attributed predominantly to the underlying severe ischaemic cardiomyopathy, and stage IV chronic kidney disease, with an uncertain contribution from the concurrent functional mitral regurgitation (due to body habitus and difficult transthoracic imaging, the MR was initially determined as moderate). Coronary angiography was not performed given recent arterial revascularization, lack of angina pectoris, and borderline kidney function at risk for contrast nephropathy.

Pertinent laboratory tests (on transfer for transcatheter edge to edge repair—TEER) included B-natriuretic peptide (BNP) level of 1.328 pg/mL (<100 pg/mL), INR 1.44 (0.80–1.20), albumin 2.9 g/dL (3.4–5.0 g/dL), total bilirubin 1.8 mg/dL (0.3–1.2 mg/dL), gGT 114 U/L (<73 U/L), urea 98 mg/dL (19–49 mg/dL), creatinine 2.3 mg/dL (0.7–1.3 mg/dL), sodium 137 mg/dL (132–146 mg/dL), Hs troponin 79 ng/L (<47 ng/L), and CK-MB 1.80 IU/L (≤5.0 IU/L).

**Figure 1:**
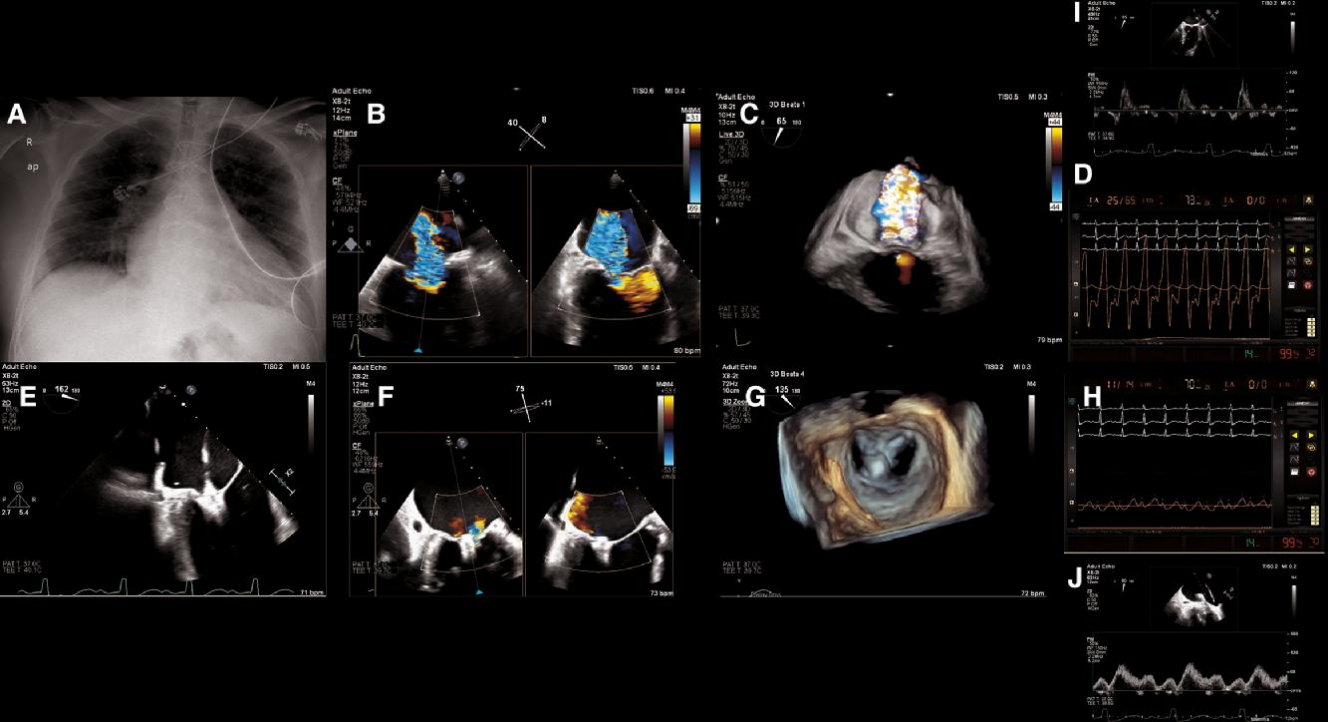
(*A*) Admission chest radiograph (posteroanterior view). Cardiomegaly with pulmonary congestion and interstitial pulmonary oedema. (*B*) Midesophageal commissural biplane view with colour flow Doppler. Wide vena contracta of mitral regurgitation measuring 32 mm. (*C*) Live 3D colour from a commissural view. Severe mitral regurgitation from across A2-P2 area. (*D*) Left atrial haemodynamic tracing upon entry. Giant V waves of 65 mmHg, indicative of severe mitral regurgitation. (*E*) Leaflet clasping with Pascal device. (*F*) Final result in biplane imaging with colour flow Doppler. (*G*) Three-dimensional enface view of the mitral valve after Pascal implantation. Double orifice valve. (*H*) Final left atrial haemodynamics after Pascal implantation. V waves of 14 mmHg. (*I*) Pulmonary venous flow before TEER. Systolic flow reversal. (*J*) Pulmonary venous flow post-TEER. Systolic S wave predominance.

A chest radiograph revealed cardiomegaly and interstitial pulmonary oedema (*Figure 1A*).

Transthoracic (difficult study due to obesity) and ultimately transoesophageal echocardiography (TOE) revealed a dilated left ventricle (end-diastolic diameter 72 mm), with an estimated left ventricular ejection fraction (LVEF) of 20% while on continuous dobutamine infusion (see Supplementary material online, *Video 1*). A severe, centrally directed MR jet (evident on TOE), originating from across the coaptation zone in the A2-P2 area, was attributed to annular dilatation and decreased leaflet mobility in systole (Carpentier types I and III-b) (*Figure 1B and C*, Supplementary material online, *Videos 2*, *3*, and *4*). The MR was considered disproportionate with an effective regurgitant orifice of 80 mm^2^ and a regurgitant volume of 76 mL. Systolic pulmonary artery pressure was 60 mmHg.

A right heart catheterization for confirmation of the adverse haemodynamics was considered, but due to the presence of left bundle branch block (LBBB), and the unstable clinical status, this was not performed.

The calculated Society for Thoracic Surgery mortality for mitral valve replacement was estimated at 9.48% with a morbidity/mortality score of 40.4%. The Heart Team decision was to proceed with mitral TEER using the Pascal P10 (Edwards Lifesciences) in an attempt to treat the pathology with a single device (width of 10 mm). Usage of the Wide MitraClip G4 or of the Pascal Ace (width of 6 mm) might have required positioning multiple devices for an optimal result increasing procedural complexity.

Left atrial haemodynamic tracings revealed giant V waves of up to 65 mmHg (mean LA pressure 32 mmHg) (*Figure 1D*). A single Pascal device (P10) was implanted in the A2-P2 area (*Figure 1E*, Supplementary material online, *Videos 5* and *6*), resulting in a double orifice valve with mild residual MR (*Figure 1F* and *G*, Supplementary material online, *Videos 7* and *8*), without stenosis (mean gradient 3 mmHg at a heart rate of 70/min). The final left atrial haemodynamics revealed V waves of 14 mmHg (mean pressure 10 mmHg) (*Figure 1H*). Changes in pulmonary vein flow were also observed (*Figure 1I* and *J*) with restoration of forward systolic flow.

The patient, as a result, had an abrupt clinical improvement allowing weaning off inotropes and was discharged on post-operative Day 4. A cardiac resynchronization and defibrillator pacemaker was electively implanted 2 weeks later.

Medical therapy could then be further up titrated and, with improved kidney function, sacubitril/valsartan was initiated. At the 1-year visit, the patient reported no rehospitalizations for heart failure, and he was in NYHA functional class II. The echocardiogram revealed minimal residual MR (see Supplementary material online, *Videos 9* and *10*), with an LVEF of 20–25%. A BNP level at 14 months post-procedure was 104 pg/mL. At the 4-year follow-up, the patient remains clinically compensated while on guideline-directed medical therapy including carvedilol 12.5 mg twice daily, sacubitril/valsartan 49/51 mg twice daily, and eplerenone 25 mg once daily.

## Discussion

Significant functional mitral regurgitation is known to adversely affect prognosis of patients with heart failure independently of LVEF.^1–3^ However, still in clinical practice, there is often uncertainty and ambivalence as to the significance and impact of functional MR. The MITRA-FR study^7^ failed to show clinical benefit of transcatheter repair of functional MR considered as severe with an ERO > 20 mm^2^, in patients with an LVEF of 15–40%. Conversely, the COAPT study^5^ that included moderate to severe (ERO 30–40 mm^2^) or severe (ERO > 40 mm^2^) functional MR in patients with an LVEF of 20–50% already on optimal guideline-directed medical therapy showed dramatic, rarely seen, improvements in the composite of death or heart failure hospitalizations with transcatheter mitral valve repair. These vastly different outcomes have been attributed to more stringent adherence to heart failure medical therapy in the COAPT study, along with the inclusion of patients with more severe levels of functional MR as per the US guidelines.^8^ Indeed a significant number of patients (52%) with moderate MR (EROA < 30 mm^2^) were enrolled in the MITRA-FR trial, whereas only 14% of patients with this parameter were enrolled in the COAPT trial.^8^ Of note, the most recent valvular heart disease guidelines^9,10^ indicate transcatheter mitral valve repair for severe functional regurgitation mainly for those patients meeting the inclusion criteria of the COAPT study and in whom surgical revascularization is not indicated.

For many patients with heart failure and underlying cardiomyopathy, the presence of significant (or disproportionate) functional MR instead of a ‘bystander’ disease actually becomes the dominant driver of symptoms and compounds the low cardiac output state leading to cardiogenic shock.^11,12^ It is in these patients that the term ‘functional’ MR becomes a misnomer, as in fact the so called ‘otherwise normal’ mitral valve is actually a severely dysfunctional valve with a wide malcoaptation zone, and haemodynamics more akin to a destroyed valve.

Transcatheter edge to edge repair using the Pascal device has been proved to be safe and effective in the treatment of mitral regurgitation.^13,14^ This device features a nitinol-based frame, an independent clasping of the leaflets, a spacer that occupies the regurgitant orifice and wide paddles that oppose the leaflets to the spacer. The dramatic improvement of both echocardiographic and haemodynamic parameters after successful M-TEER with Pascal along with the subsequent step down from the ICU setting, and discharge home soon thereafter, established the MR as the main driver of symptoms and poor clinical status in this patient, more so than the underlying cardiomyopathy.

Cardiac resynchronization therapy (CRT) was performed 2 weeks after TEER and likely also contributed to the mid-term clinical stabilization. Cardiac resynchronization therapy was well indicated given the presence of ischaemic cardiomyopathy with LBBB. However, it was delayed, initially while awaiting recovery from the surgical revascularization (performed 6 months prior), and then further delayed due to unstable haemodynamics and need for inotropic support. While CRT implantation is also feasible in the acute setting, a TEER first approach was decided by individual assessment as potentially more likely to rapidly reverse the patient’s unstable haemodynamic status.

In summary, this case exemplifies the malignant nature of severe and disproportionate functional mitral regurgitation in compounding the low cardiac output state of patients with left ventricular systolic dysfunction. Prioritizing treatment with TEER in haemodynamically unstable patients restores patient prognosis and allows up-titration of guideline-directed medical therapy and CRT to affect mid- and long-term clinical benefits.

## Patient’s perspective

A dramatic comeback. From a hopeless, ICU bound situation, with limited options, I was able to return to my home and independently continue living.

## Supplementary Material

ytae041_Supplementary_Data

## Data Availability

All data pertaining to this case report are readily available upon request.

## Lead author biography

Graduate of the University of Athens Medical School in Greece. Residency and fellowship training at St. Raphael’s Hospital, Yale New Haven Affiliate, CT, USA. Since October 2010 attending and later associate director at the Department of Transcatheter Heart Valves, Hygeia Hospital Athens, Greece. Areas of interest include interventional echocardiography and structural heart interventions.

## Supplementary material

**Consent:** Patient consented to publication of this case report for educational purposes, and for social media educational campaigns. Compliance with COPE guidelines confirmed.

**Funding:** None received.
